# Influence of pharmacogenetics on the diversity of response to statins associated with adverse drug reactions

**DOI:** 10.1515/almed-2023-0123

**Published:** 2023-10-11

**Authors:** Jaime I. Sainz de Medrano Sainz, Mercè Brunet Serra

**Affiliations:** Servicio de Bioquímica y Genética Molecular, Centro de Diagnóstico Biomédico, Hospital Clínic de Barcelona, Barcelona, Spain; Jefa de sección de Farmacología y Toxicología, Servicio de Bioquímica y Genética Molecular, Centro de Diagnóstico Biomédico, Hospital Clínic de Barcelona, Barcelona, Spain

**Keywords:** statins, pharmacogenetics, adverse reactions, *SLCO1B1*, precision medicine

## Abstract

**Background:**

Statins are one of the most prescribed medications in developed countries as the treatment of choice for reducing cholesterol and preventing cardiovascular diseases. However, a large proportion of patients experience adverse drug reactions, especially myotoxicity. Among the factors that influence the diversity of response, pharmacogenetics emerges as a relevant factor of influence in inter-individual differences in response to statins and can be useful in the prevention of adverse drug effects.

**Content:**

A systematic review was performed of current knowledge of the influence of pharmacogenetics on the occurrence and prevention of statin-associated adverse reactions and clinical benefits of preemptive pharmacogenetics testing.

**Summary:**

Genetic variants *SLCO1B1* (rs4149056) for all statins; *ABCG2* (rs2231142) for rosuvastatin; or *CYP2C9* (rs1799853 and rs1057910) for fluvastatin are associated with an increase in muscle-related adverse effects and poor treatment adherence. Besides, various inhibitors of these transporters and biotransformation enzymes increase the systemic exposure of statins, thereby favoring the occurrence of adverse drug reactions.

**Outlook:**

The clinical preemptive testing of this pharmacogenetic panel would largely prevent the incidence of adverse drug reactions. Standardized methods should be used for the identification of adverse effects and the performance and interpretation of genotyping test results. Standardization would allow to obtain more conclusive results about the association between *SLCO1B1, ABCG and CYP2C9* variants and the occurrence of adverse drug reactions. As a result, more personalized recommendations could be established for each statin.

## Introduction

Precision medicine is based on the standardized application of clinical criteria most frequently grounded in the interpretation of a set of validated biomarkers. This practice favors the use of novel preventive, diagnostic and therapeutic strategies that consider the individual characteristics of each patient. One of the goals of precision medicine is to personalize prevention and pharmacological treatment of diseases by considering factors proven to be involved in inter-individual variability. Differences in the type and severity of response may be due to different causes, including genetic (pharmacogenetics) and environmental (epigenetics) factors, treatment adherence, drug-drug interactions, physiopathological factors, and ethnicity [1, 2].

Pharmacogenetics plays an essential role in personalized medicine. It is mainly aimed at preventing the occurrence of adverse effects and improving the drug efficacy [3, 4]. Improving the profile of efficacy and safety of pharmacological treatments is especially relevant to polymedicated patients, as adverse drug reactions (ADRs) and therapeutic failure may be more prevalent in these patients.

Implementing personalized preventive and therapeutic measures for cardiovascular diseases is of paramount importance because they are the leading cause of morbimortality in developed countries, causing 874,613 deaths per year in the United States, and 4,1 million deaths in Europe in 2019, and 19.05 million deaths globally in 2020 [5, 6]. In Europe, this incidence accounts for approximately 40 % of all causes of death [6].

Apo-B100-containing lipoproteins, especially low-density lipoprotein cholesterol (LDLc), are known to be the main cause of atherogenesis [7]. Hypercholesterolemia is the primary target of cardiovascular risk reduction programs. In these programs, statin therapy for the primary prevention of cardiovascular disease (CVD) could produce a 15 % reduction in the risk for vascular death for each 38.6 mg/dL reduction of LDLc [6]. Different scientific and scholar entities, such as the American Heart Association (AHA) and the European Atherosclerosis Society (EAS), emphasize the relevance of statins in the treatment and prevention of CVD [8]. In 2018, atorvastatin and simvastatin were the most frequently prescribed drugs in the United States, ranking 1 and 10, respectively. Thus, one in four Americans aged ≥40 years receive statin therapy [9]. In Spain, according to the Spanish Agency for Medicine and Health Products (*Agencia Española de Medicamentos y Productos Sanitarios*, AEMPS), the defined daily dose per 1,000 inhabitants and day was 110 mg in 2021. The daily dose was 63.34 mg for atorvastatin and 26.67 mg for simvastatin [10].

Cholesterol is synthesized from acetyl-coenzyme A. The rate-limiting step in its synthesis is the reduction of hydroxymethylglutaryl (HMG) into mevalonate via the enzyme HMG-CoA reductase. Akira Endo hypothesized that some organisms may inhibit this enzyme as a defense mechanism against microorganisms that need cholesterol for survival [7, 11]. The first lipid-lowering agent discovered was mevastatin, isolated from *Penicillium citrinum* in 1970. Mevastatin has a similar structure to HMG-CoA and is a potent competitive inhibitor of HMG-CoA reductase [12]. Other statins were subsequently developed [11]. First-generation statins, the least effective lovastatin, pravastatin and fluvastatin, were approved in the United States by the end of the ’80s and ’90s. Second-generation statins, atorvastatin and simvastatin, were more effective in reducing LDLc. Finally, regarding the third-generation statin, rosuvastatin is the most effective lipid-lowering agent [13]. Although all statins contain the same pharmacophore group, they have different ring structures binding to the active form, which determines their chemical structure, pharmacokinetics, clinical effects or pharmacological properties, including solubility. This explains that some molecules are more hydrophilic, such as pravastatin and rosuvastatin, whereas others are more lipophilic, such as atorvastatin, lovastatin, fluvastatin, pitavastatin and simvastatin. Apparently, lipophilic molecules cause a higher prevalence of statin-associated muscle symptoms (SAMS). This could be explained by the fact that they passively cross the cell membranes of skeletal muscles and other extra-hepatic tissues, although further studies are required to validate this hypothesis [14].

Despite being a widely used pharmacological group, a considerable number of patients experience ADRs [15]. SAMS are the most frequently reported adverse reactions. This fact results in poor adherence to treatment and discontinuance, leading to increased levels of LDLc and a higher risk for CVD [8, 9, 12, 14, 16].

The association between the pharmacogenetics of statins, administered for the prevention of CVD, and the occurrence of adverse effects has been widely demonstrated. ADR vary according to the type and dose of statin [17]. The updated guidelines of the Clinical Pharmacogenomics Implementation Consortium (CPIC) published in 2022 [9] recommended *SLCO1B1*, *ABCG2* and *CYP2C9* genotyping, due to their strong association with increased systemic exposure of statins and the resulting increased risk for SAMS.

Pharmacogenetics plays a major role in personalized medicine. The use of pharmacogenetics in routine practice will guarantee that the most appropriate type and dose of statin will be selected for every patient. This review is focused on the influence of pharmacogenetics on between-subject variability of responses to these drugs and its potential role in the prevention of adverse drug reactions. A literature search was performed on PubMed of papers published between 2015 and 2023 (including some previous papers) by using the following search words: atorvastatin; simvastatin; rosuvastatin; pitavastatin; statins; pharmacogenetics; *SLCO1B1; ABCG2; CYP2C9; CYP3A4*; myopathy; myalgia; rhabdomyolysis; adverse reactions; precision medicine; meta-analysis. Special focus was placed on recommendations from the main guidelines of pharmacogenetics:CPIC and The Dutch pharmacogenomics Working Group (DPWG); and from Pharmacogenomics Knowledgebase website (PharmGKB).

## Statin pharmacology

As mentioned above, although all statins share a similar chemical structure, structural differences determine their pharmacological properties [13, 14, 18].

### Pharmacodynamics: mechanism of action and adverse drug reactions

#### Mechanism of action

**Figure 1:**
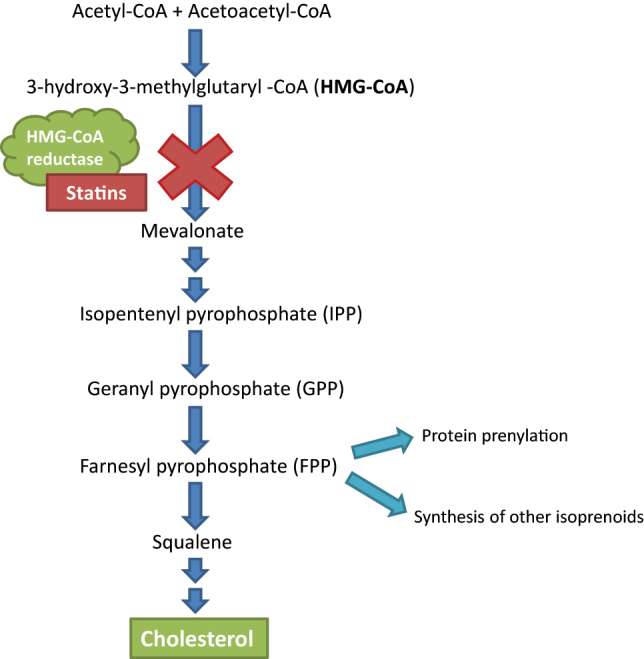
Mechanism of action of statins on the mevalonate pathway. Adapted from [11].

Statins reduce cholesterol synthesis by the liver via competitive inhibition of the enzyme HMG-CoA reductase (Figure 1) [11, 19], a rate-limiting step of its synthesis. The reduction of intracellular levels of cholesterol causes an increase in levels of LDL receptors (LDLR) in the hepatocyte. This event leads to increased LDL uptake, thereby resulting in lower plasma concentrations of LDL and other ApoB-containing lipoproteins.

The reduction of LDLc is dose-dependent and varies according to the type of statin. By average, high-intensity statins (atorvastatin 40/80 mg and rosuvastatin 20/40 mg) reduce LDLc by ≥50 %, whereas medium-intensity statis (atorvastatin 10/20 mg, rosuvastatin 5/10 mg, simvastatin 20/40 mg, pravastatin 40/80 mg, lovastatin 40/80 mg, fluvastatin 40/80 mg and pitavastatin 1/4 mg) reduce cLDL by 30–49 %. Finally, low-intensity statins reduce LDLc by <30 % (simvastatin 10 mg, pravastatin 10/20 mg, lovastatin 20 mg and fluvastatin 20/40 mg). They also cause a reduction in triglyceride levels (10–20 %). The mechanism that mediates such reduction is not fully understood, but, it may be due to an increased uptake of very low density lipoproteins (VLDL) in the hepatocyte, and a decrease in VLDL production. In addition, depending on the dose and type of statin, these drugs cause a 1–10 % increase in levels of high density lipoproteins (HDL) [20]. Statins only exert slight effects on lipoprotein (a) in plasma. Pleiotropic effects, such as antiinflammatory and antioxidant activity, have been reported *in vitro*, although their clinical relevance has not yet been demonstrated [21, 22].

Differences in the degree of binding between HMG-CoA and the different statins could explain variability in their effectiveness. Hence, atorvastatin and rosuvastatin have an additional hydrogen bond in their binding with the enzyme. Rosuvastatin also shows polar interaction with the enzyme, which could explain its increased potency [19].

#### Adverse reactions: SAMS

Statins inhibit the synthesis of cholesterol in the liver. In cases of elevated plasma concentrations, statins can also inhibit cholesterol synthesis in other extrahepatic tissues, including muscle tissue, where they can cause up to 95 % of adverse effects. Although this association and its mechanism of action have not been fully described, it is estimated that it can affect up to 30 % of patients [14]. The severity of symptoms ranges from mild to muscular disorders and potentially fatal rhabdomyolysis [17, 23].

Estimating the prevalence of SAMS is challenging, as it is based on self-reported adverse events, pain occasionally disappears, and it can be caused by a variety of factors [14, 16]. Comparison of results across studies is hindered by the lack of terminology standardization, as pain is sometimes referred to as myalgia, myositis or myopathy. A systematic review of randomized studies in patients without CVD treated with statins demonstrated the association between the occurrence of muscle symptoms (mainly weakness or stiffness) and statins. However, these symptoms do not clearly correspond to clinical muscular disorders, such as myalgia, miopathy or rhabdomyolysis [17]. On another note, a double-blind study in 12,064 patients reported an incidence of myopathy of 0.03 % in patients receiving simvastatin at low doses, and 0.9 % in those receiving simvastatin at a dose of 80 mg [14]. In agreement with these findings, Stillemans et al. [24] demonstrated the influence of the dose and systemic exposure of atorvastatin on the risk for myalgia. In contrast with these results, a meta-analysis conducted by Irwin et al., that included 192,977 patients, uncovered a slight increase in SAMS in the group of patients treated with statins. However, a dose-dependent association could not be established [25]. Finally, a meta-analysis of studies involving 18,192 elderly patients treated with atorvastatin, fluvastatin, lovastatin, pravastatin, or rosuvastatin revealed no differences in the incidence of ADRs in the treatment group, compared to the placebo group [26]. These results demonstrate some inconsistency regarding the association between statins and the occurrence of clinical muscle adverse effects.

To shed light on this problem, the PREDICTION-ADR Consortium classified the phenotype and standardized muscle injury nomenclature by establishing a scale ranging from SRM0 (statin-related myotoxicity) for mild cases, to SRM6 for severe cases [27].

Preventing muscle pain is important because it is one of the main reasons of poor adherence to or even discontinuance of treatment [27]. A variety of studies report that 50 % of patients discontinue statin therapy six months after the initiation of treatment [7, 28]. According to CPIC guidelines, SAMS affects 1–7% of patients, with a six-fold higher risk in patients treated with high doses, as compared to those receiving low doses [9]. Therefore, as recommended by the PREDICTION-ADR Consortium, it is necessary that standard terms are used in the identification and reporting of muscle-related ADRs. In addition, the development of specific biomarkers for SAMS would be clinically useful.

The use of predictive biomarkers of muscle injury, such as creatine kinase (CK), with controversial results [16], or, more recently, some miRNAs, such as miR-145 [29] or miR-499-5 [30], would be useful in the identification of SAMS.

With regard to other adverse effects, slightly elevated levels of alanine aminotransferase (ALT) have been found in 0.5–2 % of patients treated with high-intensity statins or statins at high doses, without associated hepatotoxicity [22]. Several studies have demonstrated an increase in the incidence of type 2 diabetes mellitus associated with statins at high doses and elderly patients with overweight or insulin resistance [22].

The standardization in the identification and recording of SAMS or other statin-associated adverse effects, as well as the development of specific biomarkers for these ADRs, would help to establish a clear association between the type and dose of statin and the occurrence of ADRs. Pharmacogenetic tests make it possible to identify patients at a higher risk of developing SAMS (see Section Pharmacogenetics). In any case, the clinical benefits of statins in the prevention of CVD overweigh their moderate adverse effects [17, 22].

### Pharmacokinetics and drug-drug interactions

Statins are administered orally. Chemical structure determines solubility, which affects absorption, distribution, metabolism, and excretion. Table 1 shows the pharmacokinetics of the most widely used statins [11, 12, 19, 31].

**Table 1:** Statin pharmacokinetics.

	Potency, nM^a^	Oral absorption, %	Bioavailability, %	Liver extraction, %	Binding to proteins, %	t1/2, h	Vd, L/kg	CYP450 metabolism	Renal excretion, %
Atorvastatin	1.16	30	12	70	>98	7–20	5.4	3A4 (2C8)^b^	<5
Simvastatin	1–2	60–85	<5	>80	>95	2–5	–	3A4 (2C8. 2D6)^b^	13
Rosuvastatin	0.16	50	20	63	90	20	1.7	Limited	10
Pravastatin	4	35	18	45	50	1–3	0.46	Limited	20
Lovastatin	2–4	30	5	>70	>98	2–5	–	3A4	10
Fluvastatin	3–10	98	30	>70	>98	1–3	0.42	2C9	6
Pitavastatin	0.1	80	60	?	96	10–13	0.70	Limited	–

^a^Measured as IC50 (concentration to inhibit 50 %). ^b^Minor metabolic pathway. Adapted from refs. [11, 12, 19, 31]. CYP, citochrome P; t1/2, elimination half-life; Vd, volume of distribution.

Lovastatin, simvastatin and pravastatin are derived from fungal metabolites, whereas the other statins are synthetic. Bioavailability ranges from 5 % for simvastatin and lovastatin to 60 % for pitavastatin. The bioavailability of these drugs is relatively low due to extensive first-pass uptake by the liver, which concurrently favors the pharmacological activity of statins in the liver [19].

With regard to solubility, atorvastatin, simvastatin, lovastatin, fluvastatin and pitavastatin are relatively lipophilic. Therefore, they are transported via passive diffusion, metabolized by cytochrome P450, and excreted by the biliary route [19]. Most statins, including lovastatin, simvastatin and atorvastatin, are metabolized by the CYP3A4 system, whereas fluvastatin is primarily metabolized by CYP2C9. Highly hydrophilic statins, rosuvastatin and pravastatin, require active diffusion in the liver. They are not significantly metabolized by CYP450, and they are excreted via the hepatic and renal routes [16, 19].

**Figure 2:**
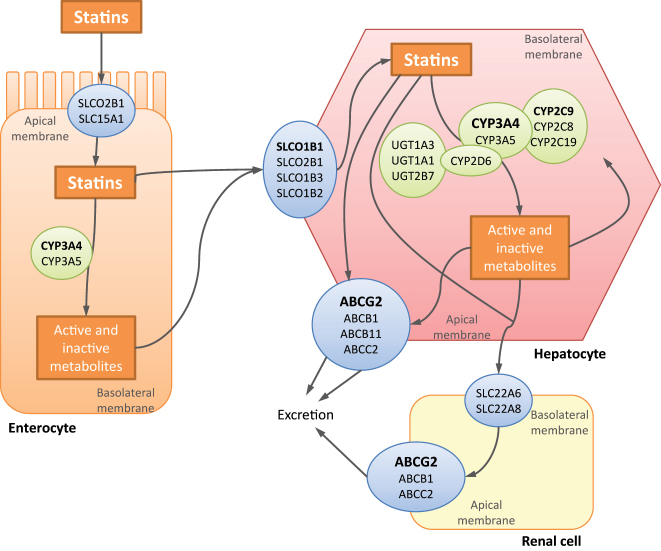
Influence of pharmacogenetics on statin distribution, exposure and effects. Figure adapted from [8, 32].

When administered in monotherapy, the incidence of statin-associated myopathy is low. However, concomitant administration of other drugs influences statin pharmacokinetics and could increase the incidence of statin-associated myopathy [9]. It has been estimated that 60 % of cases of statin-associated rhabdomyolysis are related to interaction with other drugs [16]. Statins are frequently used in combination with other drugs, due to a high proportion of patients with hyperlipidemia have other clinical conditions, such as diabetes or hypertension. The degree of drug-drug interaction is influenced by the degree of metabolization of each statin by the cytochrome P450 and their affinity for membrane transporters, such as organic anion-transporting polypeptide 1B1 (OATP1B1) or breast cancer resistance protein (BCRP) [16, 31]. Figure 2 and Supplemental Table 1 describe the transport regulatory proteins and biotransformation enzymes involved in statin distribution and exposure [9, 32]. All statins are susceptible to metabolism by CYP enzymes, with pravastatin, rosuvastatin and pitavastatin not undergoing substantial metabolism by CYP. The CYP3A4 isoenzyme is the main enzyme involved, added to CYP2C8, CYP2C9, CYP2C19 and CYP2D6. Hence, all drugs that interact with these biotransformation enzymes may increase the risk for adverse effects [14, 21, 22]. Most of these drugs are listed in Table 2.

**Table 2:** Inhibitors and inducers of biotransformation enzymes and transporter proteins that are statin substrates.

Enzyme or transport protein	Statin	Inhibitor	Inducer
CYP2C9	Fluvastatin, rosuvastatin (CYP2C19)^a^	Amiodarone, capecitabine, etravirine, fluconazole, fluvoxamine, fluvastatin, ketoconazole, metronidazole, miconazole, oxandrolone, sulfamethoxazole/trimethoprim, voriconazole, zafirlukast	Carbamazepine, phenobarbital, phenytoin, rifampicin
CYP3A4	Atorvastatin, lovastatin, simvastatin	Amiodarone, amlodipine, aprepitant, atorvastatin, bicalutamide, cilostazol cimetidine, ciprofloxacin, clarithromycin, conivaptan, cyclosporine, diltiazem, erythromycin, fluconazole, fluoxetine, fluvoxamine, grapefruit juice, imatinib, isoniazid, itraconazole, ketoconazole, mibefradil, midazolam, nefazodone, nilotinib, posaconazole, protease inhibitors, ranolazine, sertraline, tacrolimus, telithromycin, ticagrelor, tricyclic antidepressants, verapamil, voriconazole	Aprepitant, bosentan, carbamazepine, cyclophosphamide, corticosteroids, efavirenz, modafinil, nafcillin, nevirapine, phenytoin, pioglitazone, phenobarbital, rifampicin, St. John’s wort
P-gp (*ABCB1)* ^b^	Atorvastatin, lovastatin, pitavastatin, simvastatin	Amiodarone, atorvastatin, azithromycin, captopril, carvedilol, cimetidine, clarithromycin, colchicine, conivaptan, cyclosporine, diltiazem, dipyridamole, dronedarone, erythromycin, felodipine, grapefruit juice, itraconazole, ketoconazole, lovastatin, mefloquine, nicardipine, omeprazole, protease inhibitors, quinidine, ranolazine, reserpine, darolutamide sertraline, simvastatin, tacrolimus, verapamil	Carbamazepine, phenytoin, rifampicin, St. John’s wort
BCRP (*ABCG2)* ^b^	Rosuvastatin	Darolutamide	
OATP1B1 (*SLCO1B1*)^b^	Atorvastatin pitavastatin, pravastatin rosuvastatin, simvastatin	Carbamazepine, clarithromycin, cyclosporine, erythromycin, gemfibrozil, protease inhibitors, roxithromycin, rifampicin, sildenafil, sacubitril, telithromycin, glecaprevir, pibrentasvir	
OATP1B3 (*SLCO1B3*)^b^	Fluvastatin, pravastatin, rosuvastatin	Claritromicin, ciclosporin, eritromicin, rifampicin, roxitromicin rifampicin, sacubitril, telitromicin, glecaprevir, pibrentasvir	

^a^Minor metabolic pathway. ^b^Encoding gene. Adapted from refs. [8, 16, 31]. CYP, citochrome P; OATP, organic anion transporter; P-gp, glycoprotein-P.

An example of lipophilic drug is atorvastatin. Eighty-five percent of atorvastatin is metabolized by the CYP3A4 enzyme. It is also a substrate of OATP1B1, BCRP and ATP-dependent flow transporters such as the multiple drug resistance protein (MDR1) [9, 16]. The expression and activity of these transport-regulatingproteins limit the physiological effect of statins, since they determine the drug concentration that enters or is excreted from the hepatocyte. Therefore, mutations in the genes encoding these proteins are associated with changes in drug concentrations and effects (as we will discuss in Section Pharmacogenetics).

Multiple interactions have been reported between atorvastatin and drugs that are potent CYP3A4 inhibitors, including antifungal azoles (itraconazole and voriconazole); macrolides (erythromycin and clarithromycin); human immunodeficiency virus (darunavir, fosamprenavir, ritonavir, saquinavir and tipranavir) and hepatitis C virus (telaprevir) protease inhibitors, and calcium channel blockers (mibefradil). These interactions considerably increase the area under the curve (AUC) of plasma concentrations of atorvastatin.

A study of drug interactions of faldaprevir revealed that this drug increased eight times systemic exposure of atorvastatin. However, the elimination half-life of this agent decreases slightly, which suggests that this interaction results from inhibition of the hepatic uptake transporter OATP1B1 [33]. In line with this finding, the results of the studies conducted by Yamazaki et al. [34] and Alam et al. [35] demonstrated that the inhibition of the SLCO1B1 transporter by isavuconazole and chroloquine, respectively, increases exposure and the risk for SAMS.

Unlike atorvastatin, pravastin is a hydrophilic drug that is not significantly metabolized by CYP enzymes. As a result, potent inhibitors or inducers of CYP3A4, CYP2C9 or CYP2C19 will not significantly affect the pharmacokinetics of pravastin. This makes it one of the statins of choice in polymedicated patients. Its hydrophilic nature prevents it from penetrating the cell membranes of other tissues, such as muscle tissue [19].

Regarding OATP2B1 and BCRP transporters, they are chiefly expressed in enterocytes, so they can promote or attenuate absorption, respectively. Several studies have demonstrated that the concomitant treatment of pravastin and OATP1B1 inhibitors, such as cyclosporine, glecaprevir or pibrentasvir, significantly increases the AUC of pravastin in plasma [14, 36].

With regard to new biomarkers, a study on the interaction between rosuvastatin and rifampicin demonstrated that coproporfirin could be used as an endogenous biomarker of OATP1B1 inhibition. The development of biomarkers of the degree of transporter inhibition are important in assessingtheir influence on statin effects [37].

## Pharmacogenetics

For pharmacogenetics to be implemented in clinical practice, the level of evidence for the pair gene-drug association (actionable gene that supports treatment recommendations) established in different clinical guidelines should be the highest (1A). The most relevant clinical guidelines are CPIC, DPWG, PharmGKB, the Canadian Pharmacogenomics Network for Drug Safety (CPNDS), and the French National Network of Pharmacogenetics (RNPGx). The methodologies for grading scientific evidence, therapeutic recommendations based on genotype, and the level of recommendation vary according to the guideline [38, 39]. However, there are 13 genes selected in common by CPIC, DPWG, and PharmGKB, of which three have an effect on statins: *ABCG2*, *CYP2C9*, and *SLCO1B1*. Currently, definitions for *SLCO1B1* alleles comply with PharmVar standards [40].

The benefits of preemptive pharmacogenetics testing prior to treatment initiation has been widely documented and is recommended by regulatory agencies, such as the Food and Drug Administration (FDA) or the European Medicines Agency (EMA). However, their implementation in clinical practice was delayed, as entities investigated different drugs, genes or gene mutations [39]. For this reason, some European countries have selected a pharmacogenetic panel of 12 genes and 58 gene variants that enable establishing recommendations about 57 drugs, with a level of evidence of 1A, according to CPIC and DPWG guidelines [41].

This panel was evaluated in a European multicenter study. This study demonstrated that individualized therapy based on pharmacogenetic tests, according to specific drug-gene matches, reduces the incidence of ADRs and improves the clinical course of patients [42]. In February 2023, the Ubiquitous Pharmacogenomics (U-PGx) Consortium published the results of the multicenter study called Preemptive Pharmacogenomic Testing for Preventing Adverse Drug Reactions (PREPARE), conducted in seven European countries in 6,944 patients. The aim of the study was to assess the benefits of performing preemptive pharmacogenetic testing of a previously selected panel [41], known as “genetic passport”. This panel included the *CYP2C9* and *SLCO1B1* genotypes. Of note, atorvastatin was the most widely assessed drug, and a 30 % reduction was documented in clinically relevant ADRs for the drugs examined. These results demonstrate that atorvastatin therapy can be used in a cost-effective way [42].

In recent years, several studies have been conducted to assess the impact of genetic variations in biotransformation enzymes and transporters on statin pharmacokinetics and pharmacodynamics. Because in most studies, the dose-response relationship has been observed in the occurrence of toxicity with a level of evidence 1A (while the evidence level regarding efficacy is lower), polymorphisms affecting statin pharmacokinetics have been suggested to influence the occurrence and severity of adverse drug reactions [8, 9, 14, 16]. The 2022 CPIC guidelines [9] report the influence of pharmacogenetics on statin phenotype, including pharmacokinetics, SAMS, hepatotoxicity, lipid-lowering effect, and clinical efficacy. These guidelines examine a panel of genes considering the most relevant studies and opinions from experts. The genes with the highest level of evidence of their correlation with the occurrence of ADRs were *SLCO1B1* (all statins), *ABCG2* (rosuvastatin) and *CYP2C9* (fluvastatin). As a result, these guidelines provide a set of recommendations that may help reduce SAMS. Although there are reviews available on other actions such as influence on lipid-lowering effect, the guidelines only provide recommendations respect to adverse drug reactions. In relation to other genes such as *HMGCR, CYP3A4* or *CYP3A5*, although there are some ongoing studies, there is no solid evidence available that supports implementation in the clinic. Table 3 summarizes the association between the genotype (diplotypes) and transport regulatory protein function, or the prediction of the metabolizing phenotype for *SLCO1B1, ABCG2* and *CYP2C9*, respectively, based on CPIC guidelines [9].

**Table 3:** Prediction of the probable phenotype based on the *SLCO1B1, ABCG2 and CYP2C9* genotype.

Gene	Phenotype	Score	Genotype	Examples of diplotypes
*SLCO1B1*	Increased function	n/a	Carrier of two increased function alleles	*14/*14
	Normal function	n/a	Carrier of two normal function alleles or one normal+one increased function allele	*1/*1, *1/*14
	Decreased function	n/a	Carrier of one normal or increased function allele+one no function allele	*1/*5, 1/*15 (c.521T>C rs4149056)
	Poor function	n/a	Carrier of two no function alleles	*5/*5, *5/*15, *15/*15 (c.521T>C rs4149056)
*ABCG2*	Normal function	n/a	Carrier of two normal function alleles	c.421 C/C (rs2231142)
	Decreased function	n/a	Carrier of one normal function allele+one no function allele	c.421 C/A (rs2231142)
	Poor function	n/a	Carrier of two no function alleles	c.421 A/A (rs2231142)
*CYP2C9*	Normal metabolizer	2	Carrier of two normal function alleles	*1/*1
	Intermediate metabolizer	1.5	Carrier of a normal function allele+one decreased function allele OR	*1/*2 c.430C>T (rs1799853)^a^
		1	Carrier of a normal function allele+one no function allele OR two decreased function alleles	*1/*3, *2/*2 (c.430C>T rs1799853)^a^ (c.1075A>C rs1057910)^b^
	Poor metabolizer	0.5	Carrier of one decreased function allele+one no function allele	*2/*3 (c.430C>T rs1799853)^a^ (c.1075A>C rs1057910)^b^
		0	Carrier of two no function alleles	*3/*3 (c.1075A>C rs1057910)^b^

^a^Expressed in allele *2. ^b^Expressed in allele *3. Adapted from ref. [9]. n/a, not applicable.

Then, a description of the three genes mentioned above is provided, and how their genotyping allows us to predict the metabolizer or transporter function phenotype and treatment recommendations.

### SLCO1B1 (solute carrier organic anion transporter family member 1B1/OATP1B1 or OATP-C)

This transporter facilitates statin (and endogenous compounds such as bilirubin or 17-beta-glucuronosyl estradiol) uptake by the liver. Impaired function, genetically inherited or acquired due to the use of inhibitors, may increase systemic exposure, leading to the occurrence of SAMS. The *SLCO1B1* gene has 109 kilobases, is located on chromosome 12 (12p12.2) and, although several single nucleotide variants (SNVs) have been identified, only some variants are clinically relevant. The most common variant with the highest level of clinical evidence is c.521T>C, rs4149056, present in alleles *5 and *15. This variant is associated with increased systemic exposure to statins and the occurrence of SAMS. Ethnicity-based differences have been found in allelic frequency, being 0.02 for SLCO1B1*5 and 0.15 for SLCO1B1*15 in Europe.

Individuals carrying two increased function alleles (SLCO1B1*14/*14) have an increased function phenotype. In contrast, individuals carrying a normal allele and an increased function allele (SLCO1B1*1/*14) or two normal function alleles (SLCO1B1*1/*1) have a normal function phenotype. Finally, those who have a non-functional allele (e.g. SLCO1B1*5) and a normal or increased function allele have a decreased function phenotype. Carriers of two no-function alleles (e.g. SLCO1B1*5/*5) have a poor function phenotype [9, 43]. According to different studies, *SLCO1B1* variants only cause a slight reduction (<5 %) in the lipid-lowering effect of simvastatin, atorvastatin, lovastatin and pravastatin. A meta-analysis carried out in 2015 revealed no significant differences for *SLCO1B1* c521T>C, except for simvastatin, which effects were more significantly influenced by this variant [44]. Another meta-analysis reported that fluvastatin in *SLCO1B1* TT patients was associated with a higher reduction of total and LDL cholesterol [45]. In the same line, the study revealed a stronger lipid-lowering effect of fluvastatin in TT patients, as compared to heterozygous carriers [44].

On the other hand, several studies have provided clear evidence on the risk of toxicity, as systemic concentrations of some statins increase, thereby increasing the risk for myopathy. In a study in 59 patients receiving statin therapy and guided by SLCO1B1*5 genotype, a more pronounced decrease in LDLc and improved adherence were observed in SLCO1B1*5 carriers [8].

### ABCG2 (transporter of the superfamily of ABC transporters (ATP binding cassette), also known as BCRP transporter)

It is expressed in the liver, intestine and blood-brain barrier. This gene mediates the export of compounds into the extracellular space. The allele A is associated with a 30–40 % decrease of the protein and an increase of plasma levels of rosuvastatin. The *ABCG2* gene has 66 kilobases, and is located on chromosome 4 (4q22.1).The most widely studied variant is c.421C>A (rs2231142). Allele frequency is influenced by ethnicity, being 0.1 for the variant allele in Europe.

Carriers of one normal function allele and one decreased function allele have a decreased function phenotype, whereas carriers of two non-functional alleles have a poor function phenotype [9]. A meta-analysis including 423 patients demonstrated that allele A carriers of *ABCG2* c.421C>A showed increased rosuvastatin concentrations. As the frequency of the allele A in the Asian population is high (0.29), the FDA recommends reducing the dose in these patients [46].

### CYP2C9

Cytochrome P450 2C9 is involved in the first-pass metabolism of multiple drugs. Although about 71 allelic variants have been identified, the most widely described variants are the allele 2, CYP2C9*2 (c.430C>T; rs1799853), and the allele 3, CYP2C9*3 (c.1075A>C, rs1057910). These variants are associated with a 30–40 % and an 80 % decrease of function, respectively, which leads to increased systemic exposure of fluvastatin. In Europe, the allele frequencies are 0.13 for allele 2 and 0.07 for allele 3.

Carriers of two normal function alleles (CYP2C9*1/*1) have a normal metabolizer phenotype. Carriers of a normal allele and a decreased function allele (CYP2C9*1/*2) or a non-functional allele (CYP2C9*1/*3) and carriers of two decreased function alleles (CYP2C9*2/*2) have an intermediate function phenotype (intermediate metabolizers). Finally, carriers of a decreased function allele and a non-functional allele (CYP2C9*2/*3) or carriers of two non-functional alleles (CYP2C9*3/*3) have a poor function phenotype (poor metabolizers). In addition, these alleles are assigned a value according to their activity ranging from 0 to 1. Individuals with a score of 0–0.5 are poor metabolizers; those with a score of 1–1.5 are intermediate metabolizers; and those with a score of 2 are normal metabolizers [9].

All individuals carrying a variant in some of these three genes resulting in an ineffective metabolizer or transporter phenotype will have a higher risk for elevated systemic exposure to a specific statin and,as a result, these individuals will have a higher risk for the occurrence of SAMS, which requires a dose adjustment or change of statin.

Regarding other polymorphisms not considered in these guidelines, a study in 156 patients revealed a potential association between CYP3A5*1 and atorvastatin accumulation. However, these results need to be confirmed in future studies in independent cohorts [47].

Table 4 contains a set of therapeutic recommendations on dose adjustment for the different statins, on the basis of the phenotype previously predicted by genotyping. Data is based on CPIC guidelines [9] and meta-analyses [45, 46, 48]. Genotype testing is recommended prior to the initiation of treatment to consider recommendations about the most adequate type and dose of statin to be considered.

**Table 4:** Dosage recommendations based on phenotype and statin.

Statin	Phenotype	Implications^a^	Dosage recommendations	Level of recommendation^b^
Atorvastatin	SLCO1B1 decreased function (c.521T>C rs4149056)^c^	Increased risk for myopathy, as compared to normal function	Risk for myopathy at doses >40 mg. Consider combined therapy in these cases.	Moderate
	SLCO1B1 poor function (c.521T>C rs4149056)^c^	Increased risk for myopathy, as compared to normal and decreased function	Risk for myopathy at doses >20 mg. Consider rosuvastatin or combined therapy in these cases.	Moderate
Fluvastatin	SLCO1B1 decreased function (c.521T>C rs4149056)^c^	Increased risk for myopathy, as compared to normal function	Risk for myopathy at doses >40 mg.	Moderate
	SLCO1B1poor function (c.521T>C rs4149056)^c^	Increased risk for myopathy, as compared to normal and decreased function	Prescribe <40 mg. If a dose >40 mg is necessary, consider changing statin or a combined therapy	Moderate
	Normal CYP2C9 metabolizer	Normal exposure	Prescribe according to guidelines	Strong
	Intermediate CYP2C9 metabolizer (c.430C>T rs1799853)^c^ (c.1075A>C rs1057910)^c^	Increased risk for myopathy, as compared to normal metabolizer	Prescribe <40 mg. If a dose >40 mg is necessary, consider changing statin or a combined therapy	Moderate
	Poor CYP2C9 metabolizer (c.430C>T rs1799853)^c^ (c.1075A>C rs1057910)^c^	Increased risk for myopathy, as compared to normal or intermediate metabolizer	Prescribe <20 mg. If a dose >40 mg is necessary, consider changing statin or a combined therapy	Moderate
Lovastatin	SLCO1B1 decreased function (c.521T>C rs4149056)^c^	Increased risk for myopathy, as compared to normal function	Prescribe an alternative statin or limit dose to <20 mg	Moderate
	SLCO1B1 poor function (c.521T>C rs4149056)^c^	Increased risk for myopathy, as compared to normal and decreased function	Prescribe an alternative statin	Moderate
Pitavastatin	SLCO1B1 decreased function (c.521T>C rs4149056)^c^	Increased risk for myopathy, as compared to normal function	Risk for myopathy at doses >2 mg. Consider changing statin or a combined therapy in these cases.	Moderate
	SLCO1B1 poor function (c.521T>C rs4149056)^c^	Increased risk for myopathy, as compared to normal and decreased function	Risk for myopathy at doses >1 mg. Consider changing statin or a combined therapy in these cases.	Moderate
Pravastatin	SLCO1B1 decreased function (c.521T>C rs4149056)^c^	Increased risk for myopathy, as compared to normal function	Risk for myopathy at doses >40 mg.	Moderate
	SLCO1B1 poor function (c.521T>C rs4149056)^c^	Increased risk for myopathy, as compared to normal and decreased function	Prescribe <40 mg. If a dose >40 mg is necessary, consider changing statin or a combined therapy	Moderate
Rosuvastatin	SLCO1B1 decreased function (c.521T>C rs4149056)^c^	Increased risk for myopathy, as compared to normal function	Risk for myopathy at doses >20 mg.	Strong
	SLCO1B1 poor function (c.521T>C rs4149056)^c^	Increased risk for myopathy, as compared to normal and decreased function	Prescribe <20 mg. If a dose >20 mg is necessary, consider changing statin or a combined therapy	Moderate
	Normal ABCG2 function (c.421 C/C rs2231142)^c^	Typical risk for myopathy	Prescribe according to guidelines	Strong
	Reduced ABCG2 function (c.421 C/A rs2231142)^c^	Increased risk for myopathy, as compared to normal function	Prescribe according to guidelines	Moderate
	Poor ABCG2 function (c.421 A/A rs2231142)^c^	Increased risk for myopathy, as compared to normal and decreased function	Prescribe <20 mg. If a dose >20 mg is necessary, consider changing statin or a combined therapy	Moderate
Simvastatin	SLCO1B1 decreased function (c.521T>C rs4149056)^c^	Increased risk for myopathy, as compared to normal function	Prescribe an alternative statin or limit dose to <20 mg	Strong
	SLCO1B1 poor function (c.521T>C rs4149056)^c^	Increased risk for myopathy, as compared to normal and decreased function	Prescribe an alternative statin	Strong

^a^All cases of increased risk for myopathy are due to increased drug exposure. ^b^CPIC nomenclature. ^c^Genetic variant and reference SNP (rs). Detailed in Table 3. Adapted from ref. [9].

Supplemental Figure 1 describes dose adjustment for atorvastatin according to the phenotype. Dose adjustment recommendations for simvastatin are provided in Supplemental Table 2.

## Relevant aspects and future perspectives

Pharmacogenetics have been incorporated in routine practice in several European countries [42, 49]. Implementation models generally include previous testing of a panel of 12 actionable pharmacogens (58 alleles) validated with a high level of evidence (1A). This way, recommendations are provided for treatments with 57 drugs [41].

Advances in pharmacogenetics have been made possible by the work of experts, who helped overcome challenges to its clinical use [50]. Thus, the scientific community has provided responses and the tools necessary to: (i) identify gene-drug pairs with evidence 1A; (ii) the development of pharmacogenetic guidelines with clear recommendations for choice and adjustment of treatment; (iii) standardize allele testing methods and nomenclature; (iv) electronic pharmacogenomics reporting (compatible with hospital information systems); (v) make advances in genetic statistics or machine learning techniques; (vi) demonstrate cost-effectiveness; and (vii) provide training and education to health professionals and patients [9, 26, 49].

Initial experiences provide an opportunity to standardize and improve the testing procedure (from requesting preemptive pharmacogenetic testing to the reporting of pharmacogenetic results). This would make it possible to assess the clinical usefulness of pharmacogenetics in the selection of the most adequate drugs and doses. As a result, the incidence of ADRs would decrease significantly, and the efficacy of treatment could be improved at Primary Care level [51, 52].

The standardization of pharmacogenetics tests will improve the robustness of results and enable intercenter comparison. In addition, centers will be required to join External Quality Assurance Programs.

Further prospective multicentre studies on statin pharmacogenetics are required. The results obtained will help adequately assess the drug-drug interactions that influence CYP and OATP1B1 and BCRP transporter activity, thereby causing an increase in system exposure of statins and augmenting the risk for SAMS. More studies are also needed to examine whether monitoring statin concentrations in plasma (metabolizer phenotype) considering the genotype may contribute to a more personalized dose adjustment.

There is evidence that statin pharmacogenetic testing is cost-effective, especially when analyzing the 12-gene-drug pair panel [42, 53].

The panel currently selected for previous pharmacogenetics testing will be refined over time. This panel will be enriched with new findings on gene-drug pairs with a high level of evidence, and with the inclusion of novel alleles and haplotypes for known gene-drug pairs. Recommendations in recent guidelines are aimed at reducing the occurrence of SAMS. However, further studies are required to assess the impact of preemptive pharmacogenetic testing on treatment adherence, LDLc levels, and the risk for CVD [9].

Findings on the clinical benefits of preemptive pharmacogenetic testing (and therapeutic drug monitoring, when appropriate) should be disseminated via ongoing training of health professionals and patients.

In conclusion, in this new era of precision medicine, preemptive statin pharmacogenetic testing will provide clinical benefits by reducing the occurrence of ADRs. Finally, it is necessary that clinical data and laboratory methods are standardized to refine recommendations about personalized statin treatments.

## Supplementary Material

Supplementary Material

## References

[1] Lauschke VM, Zhou Y, Ingelman-Sundberg M (2019). Novel genetic and epigenetic factors of importance for inter-individual differences in drug disposition, response and toxicity. Pharmacol Ther.

[2] Hassan R, Allali I, Agamah FE, Elsheikh SSM, Thomford NE, Dandara C (2021). Drug response in association with pharmacogenomics and pharmacomicrobiomics: towards a better personalized medicine. Brief Bioinf.

[3] Swen JJ, Nijenhuis M, van Rhenen M, de Boer-Veger NJ, Buunk AM, Houwink EJF (2018). Pharmacogenetic information in clinical guidelines: the European perspective. Clin Pharmacol Ther.

[4] Wang L, Scherer SE, Bielinski SJ, Muzny DM, Jones LA, Black JL (2022). Implementation of preemptive DNA sequence–based pharmacogenomics testing across a large academic medical center: the Mayo-Baylor RIGHT 10K Study. Genet Med.

[5] Tsao CW, Aday AW, Almarzooq ZI, Alonso A, Beaton AZ, Bittencourt MS (2022). Heart disease and stroke statistics-2022 update: a report from the American Heart association. Circulation.

[6] Timmis A, Vardas P, Townsend N, Torbica A, Katus H, De Smedt D (2022). European society of cardiology: cardiovascular disease statistics 2021: executive summary. Eur Heart J.

[7] Toth PP, Banach M (2019). Statins: then and now. Methodist Debakey Cardiovasc J.

[8] Kitzmiller JP, Mikulik EB, Dauki AM, Murkherjee C, Luzum JA (2016). Pharmacogenomics of statins: understanding susceptibility to adverse effects. Pharmgenomics Pers Med.

[9] Cooper-DeHoff RM, Niemi M, Ramsey LB, Luzum JA, Tarkiainen EK, Straka RJ (2022). The clinical pharmacogenetics implementation consortium guideline for SLCO1B1, ABCG2, and CYP2C9 genotypes and statin-associated musculoskeletal symptoms. Clin Pharmacol Ther.

[10] Utilización de medicamentos hipolipemiantes en España Agencia Española de Medicamentos y Productos Sanitarios. Accesible desde. ..

[11] Sirtori CR (2014). The pharmacology of statins. Pharmacol Res.

[12] Kee PS, Chin PKL, Kennedy MA, Maggo SDS (2020). Pharmacogenetics of statin-induced myotoxicity. Front Genet.

[13] Zhang X, Xing L, Jia X, Pang X, Xiang Q, Zhao X (2020). Comparative lipid-lowering/increasing efficacy of 7 statins in patients with dyslipidemia, cardiovascular diseases, or diabetes mellitus: systematic review and network meta-analyses of 50 randomized controlled trials. Cardiovasc Ther.

[14] Pergolizzi JV, Coluzzi F, Colucci RD, Olsson H, LeQuang JA, Al-Saadi J (2020). Statins and muscle pain. Expert Rev Clin Pharmacol.

[15] Stroes ES, Thompson PD, Corsini A, Vladutiu GD, Raal FJ, Ray KK (2015). Statin-associated muscle symptoms: impact on statin therapy – European atherosclerosis society consensus panel statement on assessment, aetiology and management. Eur Heart J.

[16] Hirota T, Fujita Y, Ieiri I (2020). An updated review of pharmacokinetic drug interactions and pharmacogenetics of statins. Expert Opin Drug Metab Toxicol.

[17] Cai T, Abel L, Langford O, Monaghan G, Aronson JK, Stevens RJ (2021). Associations between statins and adverse events in primary prevention of cardiovascular disease: systematic review with pairwise, network, and dose-response meta-analyses. BMJ.

[18] Yebyo HG, Aschmann HE, Kaufmann M, Puhan MA (2019). Comparative effectiveness and safety of statins as a class and of specific statins for primary prevention of cardiovascular disease: a systematic review, meta-analysis, and network meta-analysis of randomized trials with 94,283 participants. Am Heart J.

[19] Schachter M (2005). Chemical, pharmacokinetic and pharmacodynamic properties of statins: an update. Fundam Clin Pharmacol.

[20] Barter PJ, Brandrup-Wognsen G, Palmer MK, Nicholls SJ (2010). Effect of statins on HDL-C: a complex process unrelated to changes in LDL-C: analysis of the VOYAGER database. J Lipid Res.

[21] Grundy SM, Stone NJ, Bailey AL, Beam C, Birtcher KK, Blumenthal RS (2019). 2018 AHA/ACC/AACVPR/AAPA/ABC/ACPM/ADA/AGS/APhA/ASPC/NLA/PCNA guideline on the management of blood cholesterol: a report of the American college of cardiology/American Heart association task force on clinical practice guidelines. Circulation.

[22] Mach F, Baigent C, Catapano AL, Koskina KC, Casula M, Badimon L (2019). 2019 ESC/EAS guidelines for the management of dyslipidaemias: lipid modification to reduce cardiovascular risk. Atherosclerosis.

[23] Bouitbir J, Sanvee GM, Panajatovic MV, Singh F, Krähenbühl S (2020). Mechanisms of statin-associated skeletal muscle-associated symptoms. Pharmacol Res.

[24] Stillemans G, Paquot A, Muccioli GG, Hoste E, Panin N, Åsberg A (2022). Atorvastatin population pharmacokinetics in a real-life setting: influence of genetic polymorphisms and association with clinical response. Clin Transl Sci.

[25] Irwin JC, Khalesi S, Fenning AS, Vella RK (2018). The effect of lipophilicity and dose on the frequency of statin-associated muscle symptoms: a systematic review and meta-analysis. Pharmacol Res.

[26] Zhou Z, Albarqouni L, Curtis AJ, Breslin M, Nelson M (2020). The safety and tolerability of statin therapy in primary prevention in older adults: a systematic review and meta-analysis. Drugs Aging.

[27] Wei MY, Ito MK, Cohen JD, Brinton EA, Jacobson TA (2013). Predictors of statin adherence, switching, and discontinuation in the USAGE survey: understanding the use of statins in America and gaps in patient education. J Clin Lipidol.

[28] Zhang H, Plutzky J, Skentzos S, Morrison F, Mar P, Shubina M (2013). Discontinuation of statins in routine care settings, A cohort study. Ann Intern Med.

[29] Saito S, Nakanishi T, Shirasaki Y, Nakajima M, Tamai I (2017). Association of miR-145 with statin-induced skeletal muscle toxicity in human rhabdomyosarcoma RD cells. J Pharm Sci.

[30] Min P-K, Park J, Isaacs S, Taylor BA, Thompson PD, Troyanos C (2016). Influence of statins on distinct circulating microRNAs during prolonged aerobic exercise. J Appl Physiol.

[31] Wiggins BS, Saseen JJ, Page RL, Reed BN, Sneed K, Kostis JB (2016). Recommendations for management of clinically significant drug-drug interactions with statins and select agents used in patients with cardiovascular disease: a scientific statement from the American Heart association. Circulation.

[32] Guan ZW, Wu KR, Li R, Yin Y, Li XL, Zhang SF (2019). Pharmacogenetics of statins treatment: efficacy and safety. J Clin Pharm Ther.

[33] Huang F, Marzin K, Koenen R, Kammerer KP, Strelkowa N, Elgadi M (2017). Effect of steady-state faldaprevir on pharmacokinetics of atorvastatin or rosuvastatin in healthy volunteers: a prospective open-label, fixed-sequence crossover study. J Clin Pharmacol.

[34] Yamazaki T, Desai A, Goldwater R, Han D, Lasseter KC, Howieson C (2017). Pharmacokinetic interactions between isavuconazole and the drug transporter substrates atorvastatin, digoxin, metformin, and methotrexate in healthy subjects. Clin Pharmacol Drug Dev.

[35] Alam K, Pahwa S, Wang X, Zhang P, Ding K, Abuznait AH (2016). Downregulation of organic anion transporting polypeptide (OATP) 1B1 transport function by lysosomotropic drug chloroquine: implication in OATP-mediated drug-drug interactions. Mol Pharm.

[36] Yee SW, Giacomini MM, Shen H, Humphreys WG, Horng H, Brian W (2019). Organic anion transporter polypeptide 1B1 polymorphism modulates the extent of drug–drug interaction and associated biomarker levels in healthy volunteers. Clin Transl Sci.

[37] Lai Y, Mandlekar S, Shen H, Holenarsipur VK, Langish R, Rajanna P (2016). Coproporphyrins in plasma and urine can be appropriate clinical biomarkers to recapitulate drug-drug interactions mediated by organic anion transporting polypeptide inhibition. J Pharmacol Exp Ther.

[38] Abdullah-Koolmees H, van Keulen AM, Nijenhuis M, Deneer VHM (2021). Pharmacogenetics guidelines: overview and comparison of the DPWG, CPIC, CPNDS, and RNPGx guidelines. Front Pharmacol.

[39] Waqas A, Chen L, Tagwerker C, Alshabeeb MA (2022). Pharmacogenes that demonstrate high association evidence according to CPIC, DPWG, and PharmGKB. Front Med.

[40] Ramsey LB, Gong L, Lee S-B, Wagner JB, Zhou X, Sangkuhl K (2023). PharmVar GeneFocus: SLCO1B1. Clin Pharmacol Ther.

[41] van der Wouden CH, Bank PCD, Özokcu K, Swen JJ, Guchelaar HJ (2019). Pharmacist-initiated pre-emptive pharmacogenetic panel testing with clinical decision support in primary care: record of PGx results and real-world impact. Genes.

[42] Swen JJ, van der Wouden CH, Manson LE, Abdullah-Koolmees H, Blagec K, Blagus T (2023). A 12-gene pharmacogenetic panel to prevent adverse drug reactions: an open-label, multicentre, controlled, cluster-randomised crossover implementation study. Lancet.

[43] Ramsey LB, Johnson SG, Caudle KE, Haidar CE, Voora D, Wilke RA (2014). The clinical pharmacogenetics implementation consortium guideline for SLCO1B1 and simvastatin-induced myopathy: 2014 update. Clin Pharmacol Ther.

[44] Dou Y, Zhu X, Wang Q, Tian X, Cheng J, Zhang E (2015). Meta-analysis of the SLCO1B1 c.521T>C variant reveals slight influence on the lipid-lowering efficacy of statins. Ann Lab Med.

[45] Xiang Q, Zhang X, Ma L, Hu K, Zhang Z, Mu G (2018). The association between the SLCO1B1, apolipoprotein E, and CYP2C9 genes and lipid response to fluvastatin: a meta-analysis. Pharmacogenet Genomics.

[46] Song Y, Lim HH, Yee J, Yoon HY, Gwak HS (2022). The association between ABCG2 421C>A (rs2231142) polymorphism and rosuvastatin pharmacokinetics: a systematic review and meta-analysis. Pharmaceutics.

[47] Zubiaur P, Benedicto MD, Villapalos-García G, Navares-Gómez M, Mejía-Abril G, Román M (2021). SLCO1B1 phenotype and CYP3A5 polymorphism significantly affect atorvastatin bioavailability. J Pers Med.

[48] Xiang Q, qing CS, yue ML, Hu K, Zhang Z, yan MG (2018). Association between SLCO1B1 T521C polymorphism and risk of statin-induced myopathy: a meta-analysis. Pharmacogenomics J.

[49] Jarvis JP, Peter AP, Keogh M, Baldasare V, Beanland GM, Wilkerson ZT (2022). Real-World impact of a pharmacogenomics-enriched comprehensive medication management program. J Pers Med.

[50] Relling MV, Klein TE (2011). CPIC: Clinical pharmacogenetics implementation consortium of the pharmacogenomics research network. Clin Pharmacol Ther.

[51] Jansen ME, Rigter T, Fleur TMC, Souverein PC, Verschuren WMM, Vijverberg SJ (2023). Predictive value of SLCO1B1 c.521T>C polymorphism on observed changes in the treatment of 1136 statin-users. Genes.

[52] Rigter T, Jansen ME, Groot JM., Janssen SWJ, Rodenburg W, Cornel MC (2020). Implementation of pharmacogenetics in primary care: a multi-stakeholder perspective. Front Genet.

[53] Brunette CA, Dong OM, Vassy JL, Danowski ME, Alexander N, Antwi AA (2021). Article a cost–consequence analysis of preemptive SLCO1B1 testing for statin myopathy risk compared to usual care. J Pers Med.

