# Supplementary material for: Influence of pharmacogenetics on the diversity of response to statins associated with adverse drug reactions
Source: Adv Lab Med. 2023 Oct 11;4(4):341–52. doi: 10.1515/almed-2023-0123 (PMC10724874; doi:10.1515/almed-2023-0123)
Supplement: Supplementary file 1 — Supplementary Material [file j_almed-2023-0123_suppl_001.docx]

| **Supplemental Table 1:** Transporter and metabolizing complex involved according to statin. | |
| --- | --- |
| **Transporter** |  |
| ABCB1 | Atorvastatin, lovastatin, pravastatin, rosuvastatin, simvastatin, pitavastatin |
| ABCC2 | Atorvastatin, lovastatin, pravastatin, rosuvastatin, simvastatin, pitavastatin |
| ABCG2 | Atorvastatin, fluvastatin, pravastatin, rosuvastatin, pitavastatin |
| ABCB11 | Pravastatin, rosuvastatin |
| SLC15A1 | Fluvastatin |
| SLC22A6 | Pravastatin |
| SLC22A8 | Pravastatin |
| SLCO1B1 | Atorvastatin, fluvastatin, lovastatin, pravastatin, rosuvastatin, simvastatin |
| SLCO2B1 | Atorvastatin, fluvastatin, lovastatin, pravastatin, rosuvastatin, simvastatin |
| SLCO1B3 | Atorvastatin, fluvastatin, lovastatin, pravastatin, rosuvastatin, simvastatin, pitavastatin |
| SLCO10A1 | Atorvastatin, lovastatin, simvastatin |
| SLCO1A2 | Rosuvastatin |
| **Metabolism** |  |
| CYP3A4 | Atorvastatin, lovastatin, simvastatin |
| CYP3A5 | Atorvastatin, lovastatin, simvastatin |
| CYP2C8 | Atorvastatin, fluvastatin, lovastatin, simvastatin |
| CYP2C9 | Atorvastatin, fluvastatin, lovastatin, rosuvastatin, simvastatin, |
| CYP2C19 | Atorvastatin, fluvastatin, lovastatin, simvastatin, |
| CYP2D6 | Atorvastatin, lovastatin, simvastatin |
| UGT1A1 | Atorvastatin, fluvastatin, lovastatin, simvastatin, |
| UGT1A3 | Atorvastatin, fluvastatin, lovastatin, simvastatin, |
| UGT2B7 | Atorvastatin, lovastatin, simvastatin |
| Adapted from [8,12] | |

| **Supplemental Table 2:** Dosage recommendations for simvastatin by SLCO1B1 phenotype | | | |
| --- | --- | --- | --- |
| **Phenotype** | **Genotype** | **Risk for myopathy** | **Recommendation** |
| Normal function, homozygous | TT: | Normal | Prescribe the desired dose and adjust based on guidelines |
| Intermediate function, heterozygous | TC (c.521T>C rs4149056)^a^ | Intermediate | Prescribe a lower dose, as compared to normal risk or consider an alternative statin |
| Poor function, homozygous | CC (c.521T>C rs4149056)^a^ | High | Prescribe a lower dose, as compared to intermediate or normal risk or consider an alternative statin |
| ^a^ Gene variant and *reference SNP* (rs) in *SLCO1B1*5* y *SLCO1B1*15*  Adapted from [8] | | | |


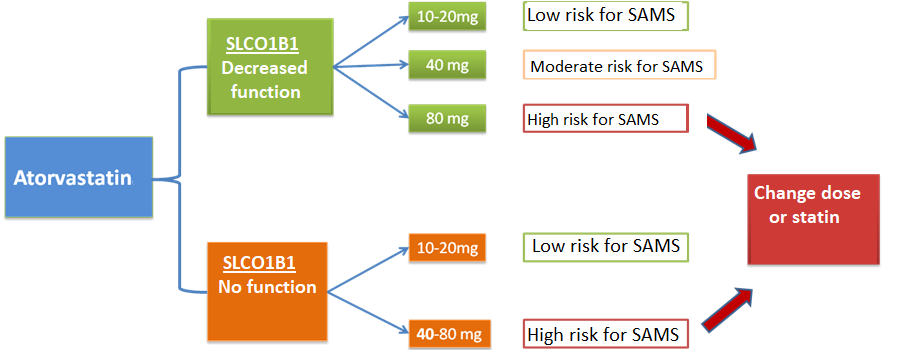


**Supplemental Figure 1.** Atorvastatin dose adjustment by phenotype. Adapted from [9].
